# Exercise for Consumptives

**Published:** 1858-10

**Authors:** 


A Special Exercise for Consumptives.*

Dr. Henry G. Davis, of New York, proposes a special exercise for consumptive patients, which, as he presumes, takes the better place of common walking exercise, or forced voluntary inspiration. In the exercises which he recommends, he makes the hands the fixed point, moving the body towards them, or suspending a part or whole of the weight by them. In this way, as he supposes, more or less of the weight of the body is thrown upon the pectoral muscles, and as they arise from the sternum and ribs, they act upon them in the same direction that they do in voluntary inspirations, without the fatigue that follows that effort. At the same time, involuntary respiration goes on without being disturbed. The author considers that it is immaterial what the exercise is, so long as the hands are made the fixed point. The patient may suspend himself by the hands from a bar, climb ropes by the hands,

* Special Exercises in the Treatment of Diseases of the Lungs. By HENRY G. DAVIS, M.D. New York: 1858.

swing by hand suspension, or if feeble, lean on some object with his hands at arms length, the object being of such a height that the body will be inclined at an angle of thirty-five or forty degrees; in which position the pectoral muscles will support a portion of the weight, and thus enlarge the chest. To secure the full advantages to be derived from this mode of exercise, it should be prosecuted faithfully. When commenced, it should be repeated every half-hour until a considerable degree of fatigue is produced.

We describe Dr. Davis's plan as one which may be useful on wet days, but as very inferior to walking exercise, when the weather invites out of doors.

